# Pulmonary vein perforation into the respiratory tract with systemic air embolism: a rare complication of left atrial appendage closure

**DOI:** 10.1186/s12890-023-02634-x

**Published:** 2023-09-28

**Authors:** Zhiqing Qiao, Liang Zhao, Bin Xu, Zhiguo Zou, Fuyu Cheng, Zien Zhou, Yuquan Xie, Jun Pu

**Affiliations:** 1 Department of Cardiology, Renji Hospital, School of Medicine, Shanghai Jiaotong University, 160 Pu Jian Road, Shanghai, 200127 China; 2 Department of Radiology, Renji Hospital, School of Medicine, Shanghai Jiaotong University, 160 Pu Jian Road, Shanghai, 200127 China

**Keywords:** Pulmonary vein, Anatomical variation, Perforation, Air embolism, Left atrial appendage closure, Complications

## Abstract

**Background:**

Pulmonary vein perforation is an uncommon complication during cardiac intervention. We present a rare case of pulmonary vein perforation into the respiratory tract with systemic air embolism during left atrial appendage closure (LAAC).

**Case presentation:**

A 77-year-old man with persistent nonvalvular atrial fibrillation was referred for percutaneous LAAC under local anaesthesia (CHA_2_DS_2_-VASc score of 4, HAS-BLED score of 3, and prior ischaemic stroke). During the procedure, after delivering a super-stiff guidewire into the left superior pulmonary vein (LSPV), the patient suddenly developed a severe cough with haemoptysis upon advancement of a delivery sheath along the guidewire. Fluoroscopy showed signs of blood entering the left main bronchus, and fast transthoracic echocardiography revealed bubbles in the left heart without pericardial effusion. The procedure was terminated because of a major complication indicated by the repeated haemoptysis and headache, and haemostatic drugs were immediately administered. Subsequent chest computed tomography angiography (CTA) revealed a filling defect in the LSPV branches and bubbles in the aorta. The patient was transferred to the critical care unit for haemostasis and antibacterial treatment. Transthoracic echocardiography later that day showed no bubbles in the heart. The headache and haemoptysis significantly abated the following day. The bubbles in the aorta disappeared on chest CTA 7 days later.

**Conclusions:**

Interventional cardiologists should pay attention to anatomical variations of the pulmonary vein, which are associated with a high risk of complications of pulmonary vein perforation during LAAC. Preoperative CTA examination and intraoperative transoesophageal echocardiography might be helpful to avoid this complication.

**Supplementary Information:**

The online version contains supplementary material available at 10.1186/s12890-023-02634-x.

## Background

Percutaneous left atrial appendage closure (LAAC) has been increasingly applied as a new nonpharmaceutical therapy for stroke prevention in patients with nonvalvular atrial fibrillation (AF) who are ineligible or fail to tolerate long-term oral anticoagulation [1]. Pulmonary artery or pulmonary vein perforation is an uncommon complication during cardiac intervention [2–4]. To date, no cases have been reported regarding pulmonary vein perforation relevant to LAAC. Herein, we present a rare case of pulmonary vein perforation into the respiratory tract with systemic air embolism during LAAC.

## Case presentation

**Fig. 1:**
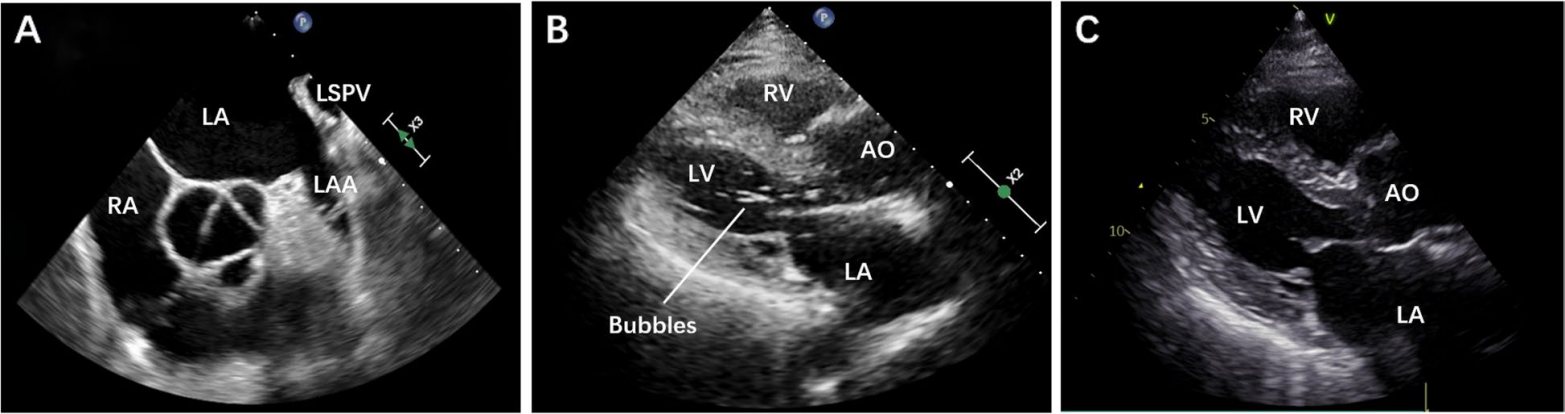
Preoperative transoesophageal echocardiography and intraoperative and postoperative transthoracic echocardiography. **A** Normal ostium of the LSPV on preoperative transoesophageal echocardiography. **B** Bubbles in the left heart on intraoperative transthoracic echocardiography. **C** No bubbles in the left heart on postoperative transthoracic echocardiography. LSPV = left superior pulmonary vein; LA = left atrium; RA = right atrium; LAA = left atrial appendage; RV = right ventricle; LV = left ventricle; AO = aorta

A 77-year-old man was admitted to our hospital with persistent nonvalvular AF for 6 years. Physical examination showed an irregular heartbeat with confirmation of AF on an electrocardiogram. Transthoracic echocardiography (TTE) revealed left and right atrial enlargement and a preserved left ventricular ejection fraction (LVEF) (58%). The patient had a CHA2DS2-VASc score of 4 and a HAS-BLED score of 3; he also had a history of ischaemic stroke and gastrointestinal bleeding, and therefore could not tolerate long-term oral anticoagulation treatment. Thus, LAAC was recommended after confirming that there was no thrombus in the left atrium (LA) and left atrial appendage (LAA) on transoesophageal echocardiography (TEE) (Fig. 1A).

**Fig. 2:**
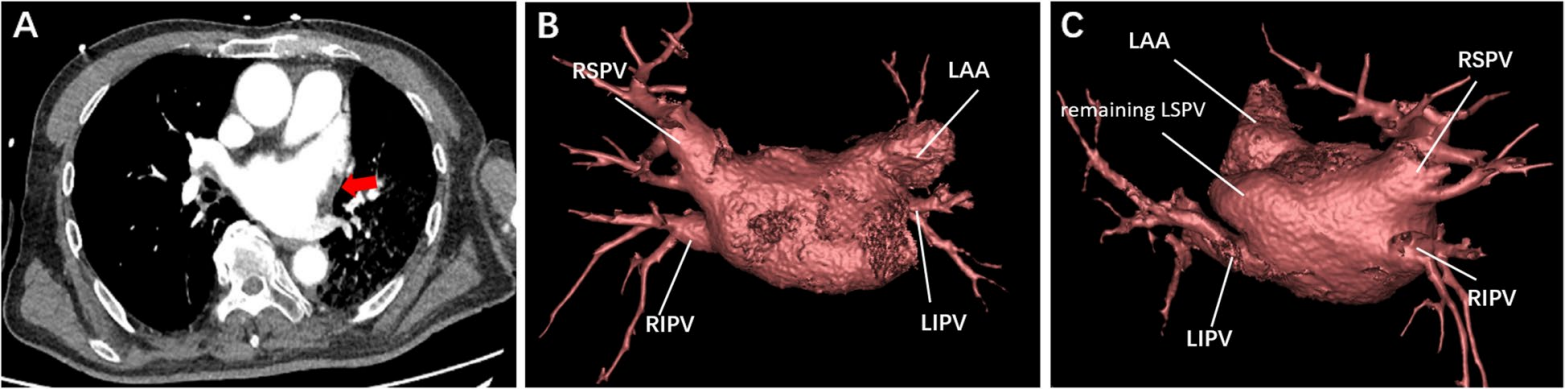
Absence of LSPV branches on postoperative chest CTA. **A** Filling defect in LSPV branches on CTA (red arrow). **B** Absence of LSPV on three-dimensionally reconstructed chest CTA (anterior view of left atrium).** C** Remaining main trunk of LSPV on three-dimensionally reconstructed chest CTA (posterior view of left atrium). CTA = computed tomography angiography; RSPV = right superior pulmonary vein; RIPV = right inferior pulmonary vein; LSPV = left superior pulmonary vein; LIPV = left inferior pulmonary vein; LAA = left atrial appendage

**Fig. 3:**
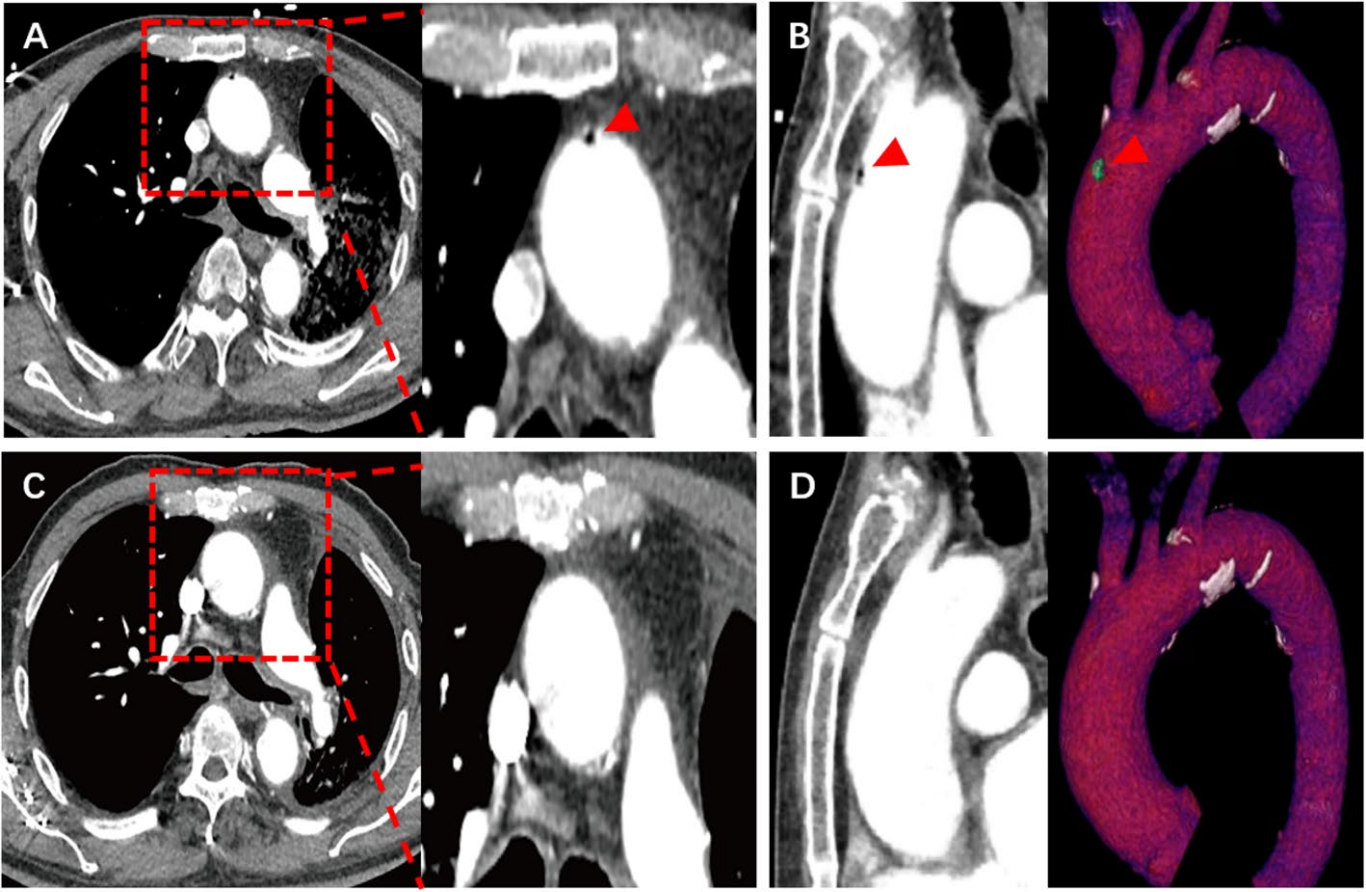
Bubbles in aorta on postoperative chest CTA. **A** Bubbles in the aorta on raw chest CTA (red arrow). **B** Bubbles in the aorta on three-dimensionally reconstructed chest CTA (red arrow). CTA = computed tomography angiography

The LAAC procedure was performed under local anaesthesia. After TTE-guided trans-septal puncture was completed, 6000 international units (IU) (100 IU/kg) of heparin was administered to achieve a minimum activated clotting time of ≥ 250 s. Next, a 0.035-inch J-tipped soft guidewire was advanced via the trans-septal puncture sheath into the left superior pulmonary vein (LSPV), followed by the Swartz sheath being delivered to the LA. The 0.035-inch J-tipped soft guidewire was then exchanged for a super stiff wire, which was placed into the LSPV while the Swartz sheath was withdrawn (Video 1). Subsequently, the patient suddenly developed a severe cough with haemoptysis when the operator began to advance the delivery sheath along the super stiff guidewire. Meanwhile, fluoroscopy showed signs of blood entering the left main bronchus (Video 2). Fast TTE identified no pericardial effusion, but bubbles were present in the LA and left ventricle (Fig. 1B, Video 3). The patient subsequently developed a headache. The procedure was terminated because of a major complication indicated by the patient’s repeated haemoptysis and headache. Protamine and etamsylate were immediately administered for haemostasis. Subsequent chest computed tomography angiography (CTA) revealed 1) absence of LSPV branches and presence of the LSPV main trunk (Fig. 2A–C) and 2) bubbles in the aorta (Fig. 3A, B). Therefore, there was a high index of suspicion of iatrogenic LSPV perforation by the super stiff guidewire with respiratory tract involvement during the procedure.

**Fig. 4:**
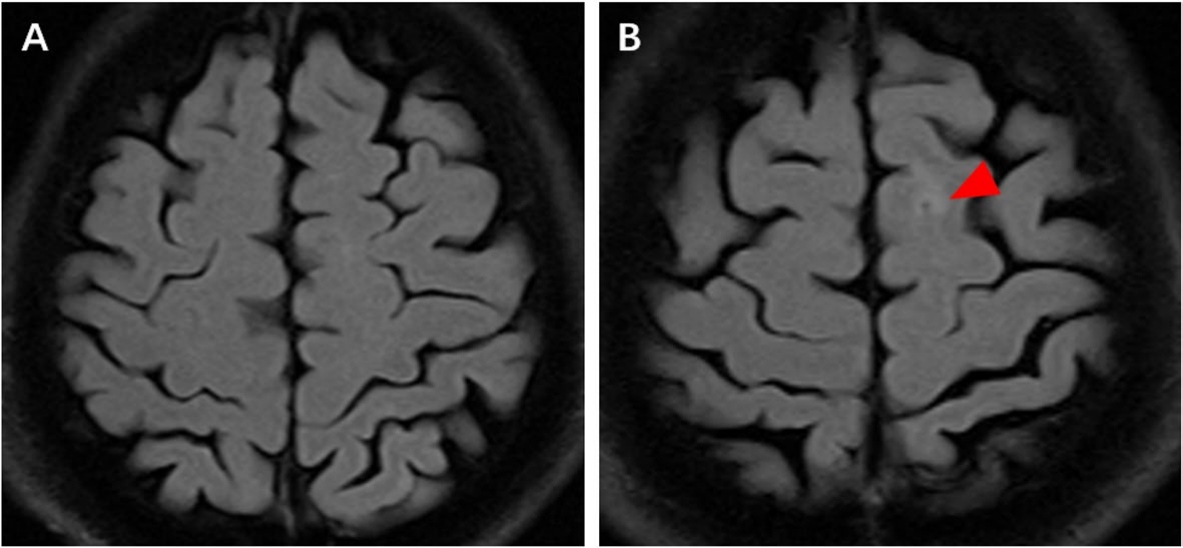
Postoperative brain MRI. **A** Absence of acute infarct lesions on postoperative brain MRI. **B** A probable microembolisation lesion in the left frontal lobe on brain MRI 7 days postoperatively (red arrow). MRI = magnetic resonance imaging

The patient was transferred to the critical care unit for haemostasis and antibacterial treatment. Postoperative brain magnetic resonance imaging showed no acute infarct lesion (Fig. 4A). TTE later that day showed no bubbles in the heart (Fig. 1C, Video 4). The headache and haemoptysis significantly abated the following day. Chest CTA on day 7 showed no bubbles in the aorta (Fig. 3C, D). However, microembolisation in the left frontal lobe was suspected on brain magnetic resonance imaging (Fig. 4B). The patient recovered well and was discharged on day 11 with neither haemoptysis nor headache.

## Discussion

According to the USA registry data from 2015 to 2017, major complications of LAAC with the Watchman™ included pericardial tamponade (1.02%), pericardial effusion (0.29%), device-related thrombosis (0.24%), and procedure-related stroke (0.08%) [5]. In addition, LAA perforation by the occluder device was a devastating complication; all three patients (0.06%) in whom this occurred died soon afterward.

Pulmonary vein perforation is an uncommon complication during cardiac intervention. Previous studies have shown that pulmonary vein perforation can be caused by catheter ablation using the cryoballoon ablation system [4, 6]. To our knowledge, there are no previous reports of pulmonary vein perforation during LAAC. We herein summarise some lessons from this case.

First, the pulmonary vein perforation in this case was caused by injury of the distal pulmonary vein branch due to an undetected atresia. Pulmonary vein atresia can be classified into congenital and acquired types that may be located proximally or distally to the pulmonary vein ostium [7–9]. Proximal pulmonary vein atresia can be detected on pre-procedural TEE; however, in this patient, TEE failed to detect the distal atresia as it was beyond the scope of TEE scanning. Distal anatomical variations of the pulmonary vein can be detected by preoperative CTA, which may prevent this complication. The recently developed image fusion technique that combines preoperative CT angiography with intraoperative fluoroscopy improves the safety of LAAC by providing real-time visualisation of cardiac structures (LA, LAA, and LSPV) during the procedure [10–12].

Second, placing the super stiff guidewire into the LSPV is a standard step of LAAC. Our procedure followed the consensus of Chinese interventional cardiologists that recommends a simplified LAAC workflow [12–14]. This includes transthoracic echocardiography-guided atrial septal puncture, no requirement for TEE monitoring throughout the procedure, and an option to use local anaesthesia because of its advantages of a shorter operation time, quicker recovery, and less harm than general anaesthesia in the older adult population. In our centre, to minimise the intolerance caused by continuous TEE monitoring under local anaesthesia, TEE was only used preoperatively to assess the left atrial appendage, and transiently applied intraoperatively to assess the sealing effects before and after occluder release. We performed the trans-septal puncture and advanced the guidewire into the pulmonary veins without TEE guidance. According to the SCAI/HRS expert consensus statement on transcatheter LAAC, intraoperative TEE or ICE assessment is recommended for the LAAC procedure [15]. TEE or ICE guidance during the procedure should reduce the risk of pulmonary vein perforation, especially when there are abnormalities of the left atrium and its surrounding structures.

**Fig. 5:**
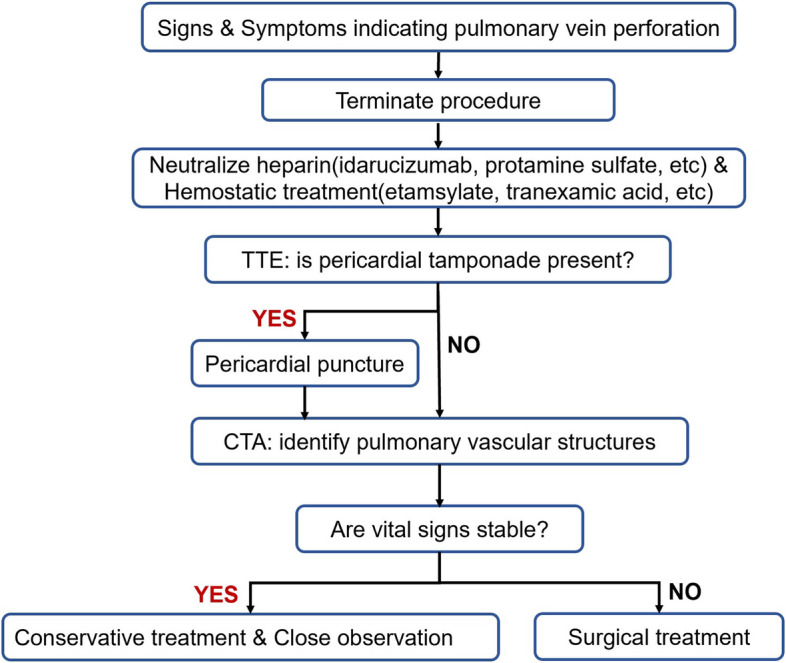
Flowchart to guide the management of patients with suspected pulmonary vein perfusion during the LAAC procedure. TTE = transthoracic echocardiography; CTA = computed tomography angiography; LAAC = left atrial appendage closure

Finally, our local anaesthesia scheme enabled us to quickly detect the patient’s symptoms (i.e., cough and haemoptysis) once the perforation occurred, and fast TTE immediately revealed bubbles in the left ventricle. Rapid diagnosis and treatment avoided unfavourable outcomes. After administration of protamine to neutralise heparin, discontinuation of anticoagulant treatment, and use of haemostatics, the haemoptysis improved with no need for further thoracotomy. If general anaesthesia had been administered, this complication would have been noted later, increasing the risk of asphyxia. Timely identification of immediate haemostasis is the key to avoiding unfavourable outcomes. We have made a flowchart of pulmonary vein perforation management based on the existing literature [4, 16–18] and our centre's experience (Fig. 5).

## Conclusions

Anatomical variations of the pulmonary vein are uncommon during LAAC, but it should be given enough attention by interventional cardiologists. Precise preoperative evaluation of anatomical variations of the pulmonary vein and utilisation of intraoperative TEE might be helpful to avoid pulmonary vein perforation. Fast TTE plays a significant role in rapid diagnosis of this complication during LAAC.

### Supplementary Information

**Additional file 1: Video 1.** Fluoroscopy of delivering a super-stiff guidewire into LSPV. A super-stiff guidewire was delivered into LSPV via the puncture sheath. LSPV= left superior pulmonary vein.

**Additional file 2: Video 2.** Fluoroscopy of blood entering the left main bronchus. The signs of blood entering the left main bronchus on fluoroscopy (red arrows) after the super-stiff wire reached LSPV. Meanwhile, the patient presented with repeated cough and haemoptysis. LSPV= left superior pulmonary vein.

**Additional file 3: Video 3.** Transthoracic echocardiography during the procedure. Bubbles (red, long arrow) in the left heart on transthoracic echocardiography during LAAC. RV=right ventricle; LV=left ventricle; LA=left atrium; AO=aorta; LAAC= left atrial appendage closure.

**Additional file 4: Video 4.** Transthoracic echocardiography after the procedure. No bubbles in the left heart on transthoracic echocardiography after LAAC. RV=right ventricle; LV=left ventricle; LA=left atrium; AO=aorta; LAAC= left atrial appendage closure.

## Data Availability

The datasets used and/or analysed during the current study are available from the corresponding author on reasonable request.
